# Speech error and tip of the tongue diary for mobile devices

**DOI:** 10.3389/fpsyg.2015.01190

**Published:** 2015-08-13

**Authors:** Michael S. Vitevitch, Cynthia S. Q. Siew, Nichol Castro, Rutherford Goldstein, Jeremy A. Gharst, Jeriprolu J. Kumar, Erica B. Boos

**Affiliations:** ^1^Spoken Language Laboratory, Department of Psychology, University of Kansas, Lawrence, KS, USA; ^2^Information Technology, University of Kansas, Lawrence, KS, USA

**Keywords:** speech error, slip of the tongue, slip of the ear, tip of the tongue, diary, mobile app

## Abstract

Collections of various types of speech errors have increased our understanding of the acquisition, production, and perception of language. Although such collections of naturally occurring language errors are invaluable for a number of reasons, the process of collecting various types of speech errors presents many challenges to the researcher interested in building such a collection, among them a significant investment of time and effort to obtain a sufficient number of examples to enable statistical analysis. Here we describe a freely accessible website http://spedi.ku.edu that helps users document slips of the tongue, slips of the ear, and tip of the tongue states that they experience firsthand or observe in others. The documented errors are amassed, and made available for other users to analyze, thereby distributing the time and effort involved in collecting errors across a large number of individuals instead of saddling the lone researcher, and facilitating distribution of the collection to other researchers. This approach also addresses some issues related to data curation that hampered previous error collections, and enables the collection to continue to grow over a longer period of time than previous collections. Finally, this web-based tool creates an opportunity for language scientists to engage in outreach efforts to increase the understanding of language disorders and research in the general public.

An experiment is the only research method that enables a scientist to establish a causal relationship between an independent and a dependent variable, but there is still much that can be learned about human behavior from naturalistic observation. Indeed, it is in the real world that a person often observes a phenomenon that intrigues and captivates that individual, leading her or him to generate a hypothesis about that phenomenon, and then to test that hypothesis (often with an experiment). Given the important role that naturalistic observation plays in the scientific method, we developed a freely-available tool accessible via the Internet to encourage expert-scientists, scientists-in-training, and science-enthusiasts to engage in the naturalistic observation of spoken language, focusing on various types of speech errors. This website amasses those observations, and makes the archive of observations available to others for further analysis.

The thorough history of the field of psycholinguistics reported in Levelt (2013) shows that the use of diaries to record naturally occurring observations has a long history in the study of *language development*. In the *production* of words and sounds, the use of diaries, especially collections of speech errors such as malapropisms and slips of the tongue, has waxed and waned over time, starting with the ground-breaking work of Meringer and Meyer (1895), with periodic resurgences in the use of diaries/collections of errors appearing in the work of Boomer and Laver, Cutler, Hockett, Fromkin, Fry, MacKay, Nooteboom, Shattuck-Hufnagel, Stemberger, and others. In contrast, diaries and collections of misperceptions, known as *slips of the ear*, have been used much less often in the study of speech *perception*; the work of Bond (1999) stands out as one of the few examples of this approach being used for several decades to study speech perception.

Despite the important role that analysis of errors can play in theories of language processing the area of speech production has one class of models that tends to focus on chronometric aspects of speech production, rather than speech errors (e.g., Levelt et al., 1999; but see Section 10 of Levelt et al., 1999), and another class of models that tends to focus on speech production errors, rather than chronometric aspects of speech production (e.g., Dell, 1986, 1988; but see Oppenheim et al., 2010). In the case of models of spoken word recognition, none of the widely-accepted models have been used to account for perception errors/slips of the ear even though many of these models have been around for several decades, and some have undergone significant revision in that time (*NAM*: Luce and Pisoni, 1998; *PARSYN*: Luce et al., 2000; *Shortlist*: Norris, 1994; Norris and McQueen, 2008; *Cohort*: Marslen-Wilson, 1987; Gaskell and Marslen-Wilson, 1997; *TRACE*: McClelland and Elman, 1986). We hope that the use of the on-line diary described in this report will lead to significant changes in the widely-accepted models of language processing with regards to accounting for various types of speech errors.

Perceptual limitations of or biases in the observer are often raised as concerns about the conclusions drawn from collections of naturally occurring speech errors. However, several studies have compared naturally occurring errors and errors that have been elicited using various techniques in controlled-laboratory settings, and found close correspondence in the types of errors observed in the two settings (e.g., Stemberger, 1985). Thus, analyses of speech errors of various types can provide ecological validity to our theories of language processing [e.g., Fay and Cutler, 1977; Vitevitch, 2002; Vitevitch et al., 2014a; see Lambert et al. (2010) for another way to provide ecological validity to theories of language processing], or as Norman (1981, p. 13) reminds us: “By examining errors, we are forced to demonstrate that our theoretical ideas can have some relevance to real behavior.”

The more significant challenges to the use of diaries to accumulate various types of naturally occurring speech errors are instead of a practical nature. First, speech errors occur “in the wild” infrequently. Wijnen (1992) estimated that adults may commit a speech error once every 1000 words produced, whereas young children may make from 4 to 8 speech errors per every 1000 words produced. Using laboratory-based techniques to elicit speech—such as tongue twisters (Shattuck-Hufnagel, 1992), Spoonerisms of Laboratory Induced Predisposition (SLIPs; Baars, 1992), or tip of the tongue (ToT) elicitation tasks (Brown and McNeill, 1966)—also tends to yield low rates of speech errors.

The low rate of naturally occurring speech errors means that an individual researcher must expend a significant amount of time and effort to collect a large enough sample of speech errors to enable the use of statistical analyses. For example, Jaeger (2005) documented over 4 years the speech errors made by her three children. In contrast, some laboratory-based experiments examining other phenomena in college-aged adults can be completed in less than 4 days. The amount of time and effort expended does not decrease very much if several participants instead of an individual researcher maintain a diary of the speech errors that they experience, as has been done to examine ToT states (Burke et al., 1991). Clearly, accumulating various types of speech errors requires much time and effort (sometimes on the part of many individuals).

Given the time and effort that is required to collect various types of speech errors, it is perhaps not surprising that diaries and other collections of errors become almost proprietary, and are made available to other researchers only after the primary researcher has thoroughly examined them. This practice may result in a delay ranging from years to decades before other investigators can examine the collections, assuming that the collections are even released at all instead of being lost due to the retirement or death of the primary researcher.

If a diary or other collection of errors is made available to other researchers, there are a number of issues related to the long-term curation of those data that must be considered. For example, as described at this URL, http://www.mpi.nl/resources/data/fromkins-speech-error-database/fromkins-speech-error-database-background, the speech error database compiled (originally on paper notecards) by Vicki Fromkin was converted some years later to a computer-readable format. However, that software format was no longer receiving technical support, which meant an invaluable resource could have easily been lost. Fortunately, Anne Cutler, Caroline Henton, Peter Ladefoged, Sieb Nooteboom, Carson Schutze, and Stefanie Shattuck-Hufnagel, with financial support from the Max Planck Society, arranged to have the database converted to XML format. The conversion process was carried out by Hansje Braam under the supervision of Sieb Nooteboom, and the resulting searchable database is available via the webpage for the Max Planck Institute for Psycholinguistics^1^. Although the Fromkin database of speech errors has a happy ending, there is an unknown number of other diaries and collections of errors that have not been preserved in this way, and have been lost to future researchers.

Data curation refers to the preservation of what has already been collected, but a related concern with diaries and other collections of errors is their continued growth. Typically diaries and collections of errors commence on a certain date, and then end on a certain date (i.e., the predetermined end of the study, or the retirement/death of the researcher). Such temporal constraints impose limits on the ultimate size of and hinder continued growth of the collection, which is counter-productive to statistical analysis where there is the desire for large sample sizes to satisfy various statistical assumptions.

A final concern about previous diaries and other collections of errors that we will discuss (although there are surely others) is that they have typically focused on a specific population, such as native speakers of a particular language who were free of any speech, language, or hearing disorders. Excluding individuals with speech, language, or hearing disorders (e.g., people who stutter, or people who are hard of hearing), individuals with cognitive impairments or diseases such as Alzheimer’s disease, non-native speakers of a given language, etc. makes sense from a methodological point of view in that a more homogenous sample reduces extraneous sources of variability that may obscure small, but theoretically important differences. Bowing to these methodological concerns, however, results in some unintended consequences. For example, the investigation of speech errors in each of the populations mentioned above (as well as cross-linguistic errors, and a number of other areas) is woefully underrepresented, thereby limiting our understanding of many aspects of language processing.

To address some of the issues discussed above (and a few others that we describe further below), we developed a method for individuals to document the observation of three common types of naturally occurring speech errors (made by themselves or by others)—slips of the ear, slips of the tongue, and ToT states—and for those individual errors to accumulate, be archived, and made available for dissemination. *Slips of the ear* (also known as *mondegreens*) refers to errors in which the speaker produces a word or phrase correctly, but the listener mis-hears what is said (Bond, 1999). In contrast, *slips of the tongue* include errors in which the speaker intends to say one thing, but instead produces something else (this includes the type of error known as a *malapropism*). Slips of the tongue include (but are not limited to) substituting one word or sound for another, exchanging sounds in adjacent words, or blending two or more words together. Finally, the *tip of the tongue state* refers to instances in which a speaker tries to retrieve a known word from the lexicon, but is unable to do so. The speaker may be able to retrieve some information about the word, such as its meaning (e.g., “the thing you use in a submarine to look above the water”), the first letter or sound of the word, or other words that sound like the target word (e.g., microscope, telescope), but not the intended word (e.g., periscope).

There are a variety of other language-related errors, e.g., *slips of the pen*: errors made when writing; *slips of the key*: errors made when typing; *slips of the finger*: errors made in signed languages, *slips of the dot*: errors made while using Braille (Wells-Jensen et al., 2007), etc., but the tool that we have developed, at present, focuses just on slips of the ear, slips of the tongue, and ToT states. Should the present database prove to be a success, it is possible that it could be expanded in the future to also include these other types of errors. In addition, slips of the tongue and other types of speech errors can be further categorized and sub-classified. We did not want to impose upon other researchers a particular theoretical perspective, so the present tool simply provides a way to amass the errors; researchers who download the archives are responsible for sorting, classifying, and “cleaning” the collected errors according to the criteria that they develop.

In addition to bringing three types of speech errors together in one resource, the present tool addresses a number of the issues described above. For example, instead of an individual researcher documenting the naturally occurring errors that he or she observes, or a small sample of participants keeping a diary for a specified period of time, the work of documenting various types of naturally occurring speech errors is distributed among all of the users of the on-line tool. “Crowd-sourcing” the documentation of naturally occurring speech errors in this way distributes the effort across a larger number of individuals (instead of being the burden of a single investigator) and also reduces the amount of time required to obtain a large sample of errors suitable for statistical analysis. Indeed, Dufau et al. (2011) used a smartphone application to collect data from over 4000 participants located around the world in a 4-month period. Other demonstrations of the speed, efficiency, and power of this approach include De Deyne et al. (2012), and Keuleers et al. (2015), among others.

With regards to the limited availability of diaries and collections of errors to other researchers, the documented errors amassed by the present tool will be available to anyone who creates an account on the website. One does not need to contribute to the site in order to access the errors that have been collected by others. Instead, we hope that users will be familiar with the problems associated with “free-riders” and the “tragedy of the commons,” and will contribute any errors that they observe if they download and analyze the errors that have been collected by others.

The on-line resource of speech errors described here also addresses the problem of previous diaries being limited in size and duration of the collection period. Because a large number of individuals can contribute to the error database, there is the potential for a very large number of errors to be amassed even if each person only contributes a small number of errors. Furthermore, the data are stored in a plain text format, making them accessible for potentially a long time (i.e., the concern about support for a particular software format disappearing is reduced). Moreover, there is great potential for the collection to grow over a very long period of time (and perhaps continue to grow after the developers have retired from the field and died). The continued growth of and the long duration that errors could be contributed opens the possibility for longitudinal analysis of errors on a large number of individuals (cf., the three children studied in Jaeger, 2005).

In addition to documenting the naturally occurring speech error, the present tool enables contributors to include other relevant information such as the presence of a regional dialect, a different native language, a speech, language or hearing disorder, etc. Including this information in the collection of errors offers more researchers the opportunity to analyze naturally occurring speech errors in a number of populations that are less often examined in mainstream psycholinguistic research, thereby greatly expanding our understanding of various language processes.

Finally, a number of on-line tools have been created for language scientists by language scientists (e.g., Vitevitch and Luce, 2004; Storkel and Hoover, 2010; Vitevitch et al., 2012). We certainly hope that the present tool will prove useful to language scientists in a variety of fields. Indeed, this resource offers researchers an easy and quick way to test a hypothesis before investing time and effort into designing and running a full-blown experiment, as well as a means to replicate with ecologically valid data the results they obtain from laboratory-based experiments (e.g., Vitevitch, 2002; Vitevitch et al., 2014a).

However, we also hope that the present tool will serve a broader, educational purpose as well. For example, a common homework assignment in Advanced Placement (AP) Psychology classes taught in high schools across the United States, and in Psychology of Language or Psycholinguistics classes taught in colleges and universities around the world is to document a speech error of some sort. Instead of depositing these assignments in the literal or electronic trashcan at the end of the semester, these individual class assignments could be amassed with the present tool (thereby increasing the size of the collection). Furthermore, the errors collected to date could serve as raw data that students in such classes could use to obtain practice in categorizing the errors and in performing statistical analyses.

The educational aspect of the present tool could also extend beyond the academic context by encouraging language enthusiasts and citizen-scientists to contribute to language research, and further educating the general public about language research. We hope that the widespread use of the on-line tool by language researchers (e.g., Linguists, Speech Pathologists, Audiologist, Psychologists, Engineers, etc.), instructors of college-level and AP classes, as well as by citizen scientists and language enthusiasts will be facilitated by making this on-line tool available for free. The next section contains more technical details about the on-line tool, as well as additional information about the type of information that is collected.

## Details about the Speech Error Diary (SpEDi)

The Pew Research Center reported that as of April, 2015: 64% of American adults have a smartphone, and 53% of American adults own a tablet computer (Pew Research Center Internet Project Survey, 2015). In order to capitalize on the ubiquity of these mobile devices we set out to develop a way to document naturally occurring speech errors that could be implemented on these ever-present devices. Because operating systems vary across devices, it would be financially prohibitive to create, maintain, and continually update an application that users could download to a wide variety of devices to document speech errors.

Instead, we developed a website that would prompt users through a number of questions to document various types of speech errors. Because the speech error diary is web-based the user does not have to download specialized software to their device (risking infection by a computer virus, etc.), nor do we have to continually update the software to keep it functional with the release of a new mobile operating system or update. To facilitate easy access to the questions a browser shortcut can be made to take the user to the automatic login page (or to automatically login the user) on any device with a web browser including tablets, mobile/smart phones, laptops, desktops, etc. This shortcut icon can be placed on the home (or other) screen of a mobile device, thereby giving the appearance that the SpEDi is a native app, while still capitalizing on the advantages of a web-based application. For instructions on how to add shortcuts to websites on various smartphone and tablet devices see: http://www.howtogeek.com/196087/how-to-add-websites-to-the-home-screen-on-any-smartphone-or-tablet/

**FIGURE 1:**
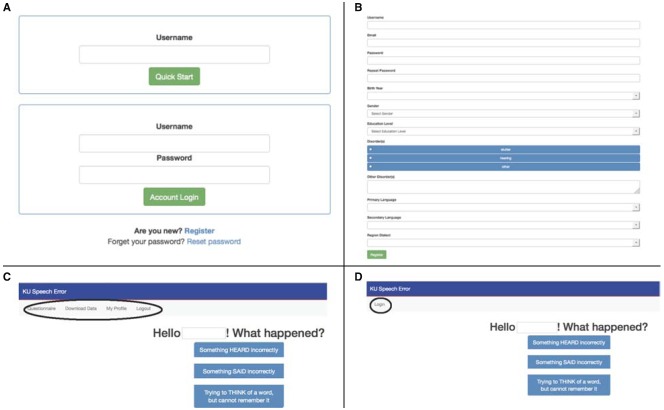
**(A)** The initial login page of the on-line speech error diary. **(B)** Information requested from new users registering with the site. **(C)** After entering the username and password using the “Account Login” option, the registered user will see the prompts that assist in documenting various types of speech errors (in the center of the screen; also accessible with the “Questionnaire” option) as well as options to “Download Data” (i.e., the errors amassed to date), to edit or update the user profile (i.e., “My Profile”), or to “Logout.” **(D)** Using the “Quick Start” option a registered user will be taken directly to the speech error prompts. Notice the slight difference in the upper left hand portion of the page when using the “Quick Start” option compared to when the “Account Login” option is used (see Figure 1C).

Below we use screen shots to help us describe how to proceed through the on-line speech error diary. As shown in panel (a) of Figure 1, the URL http://spedi.ku.edu takes the user to a webpage from which new users can register, and previously registered users can log in. New users are asked, see panel (b) of Figure 1, to generate a username and password and to provide their e-mail and minimal demographic information that will automatically populate the record for any speech error they document in themselves (if the registered user witnesses a speech error made by someone else, then the registered user will be prompted for demographic information about the speaker who made the error).

For previously registered users there are two login options. Referring back to Figure 1A, there is the “Quick Start” option, and the “Account Login” option. For the “Quick Start” option only the registered username needs to be entered, enabling the user to proceed directly to the prompts to document the observed speech error (described in more detail below). For the “Account Login” option, both the username and password must be entered. Upon doing so the user sees, as shown in Figure 1C, the first prompts to document various types of speech errors, and, importantly, also sees (in the upper left hand portion of the page) options to download the collection of errors amassed to date; the database can only be accessed by registered users through the “Account Login” option.

The “Quick Start” option was designed to quickly take registered users to the error prompts, thereby facilitating the documentation of the speech error. By comparing Figures 1C,D the reader will notice a slight difference in the upper left hand portion of the page when the “Quick Start” option is used. Most importantly, the errors amassed to date cannot be downloaded when a registered user enters the site via the “Quick Start” option, otherwise the prompts for documenting the speech error are the same, and described below. Again, to facilitate easy access to the initial page of the speech error diary, a user can use the preferred web browser to create a shortcut to the “Quick Start” page, and place the shortcut on their desktop/tablet-top/phone-top.

## Something HEARD Incorrectly

**FIGURE 2:**
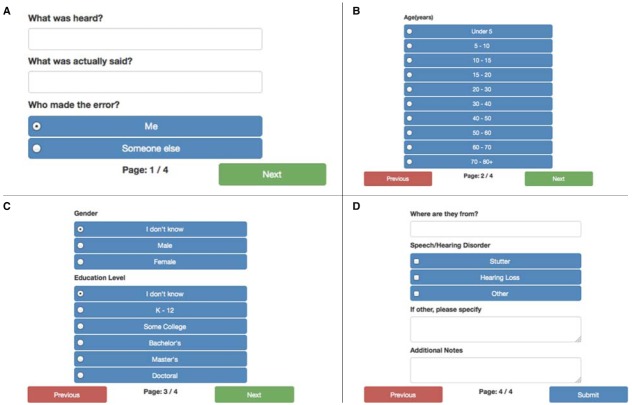
**(A)** The first screen for the “Something HEARD incorrectly” prompt. **(B)** The second screen of the “Something HEARD incorrectly” prompt and the “Something SAID incorrectly” prompt, soliciting the user for demographic information about the individual who made the error that was documented on the first screen. **(C)** The third screen of the “Something HEARD incorrectly” prompt and the “Something SAID incorrectly” prompt, soliciting the user for demographic information about the individual who made the error that was documented on the first screen. **(D)** The fourth screen of the “Something HEARD incorrectly” prompt and the “Something SAID incorrectly” prompt, soliciting the user for demographic information about the individual who made the error that was documented on the first screen.

When a slip of the ear occurs, that is the user hears something incorrectly or when the user witnesses someone else mishearing something that was said correctly, the button for “Something HEARD incorrectly” should be pressed. This leads the user to the screen displayed in Figure 2A, which prompts the user to enter in the words or phrase that was (incorrectly) heard, and then the words or phrases that were actually (correctly) spoken. The user is also asked to indicate if they were the person who misheard the words or phrases, or if they witnessed the mishearing; perhaps the user was the person who spoke correctly but their interlocutor misheard what was said, or the user was a third-party in a group where the slip of the ear occurred.

Once the error has been documented the user is then prompted for: an estimate of the age of the speaker on the second of four screens (see Figure 2B), and information about the gender and education level of the speaker on the third of four screens (see Figure 2C). On the final screen of prompts (see Figure 2D) the user is asked: “Where are they from?” which is intended to unobtrusively obtain information related to regional dialects (we thank Zinny Bond for suggesting this approach), for information related to speech, language, or hearing disorders, as well as options for any other type of cognitive or neurological disorder that might have contributed in some way to the error. There is also a prompt for “Additional notes” where a user might document that an alcoholic beverage had been consumed shortly before the error was made, that the error occurred in a noisy environment, or document any other facts that might be of interest. The user is not required to enter information on the screens depicted in Figures 2B–D, but at least some of this information would be useful for others analyzing the collection of errors in the future.

## Something SAID Incorrectly

**FIGURE 3:**
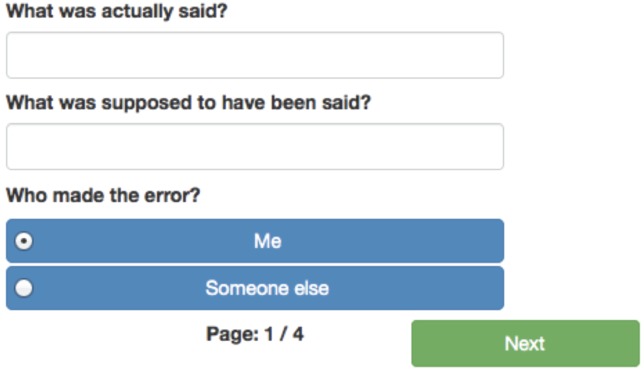
**The first screen for the “Something SAID incorrectly” prompt**.

When a slip of the tongue occurs, that is the user says something incorrectly or when the user witnesses someone else saying something incorrectly, the button for “Something SAID incorrectly” should be pressed. This leads the user to the screen displayed in Figure 3, which prompts the user to enter in the words or phrase that were incorrectly spoken (“What was actually said?”), and then the words or phrases that were intended (or that the speaker spontaneously uttered as a correction, “What was supposed to have been said?”). The user is also asked to indicate if they were the person who misspoke the words or phrases, or if they witnessed someone else making the error. Once the error has been documented the user is prompted for the same demographic information that appeared for the “Something SAID incorrectly” prompt (see again Figures 2B–D).

## Trying to THINK of a Word, but Cannot Remember it

In a ToT state a person tries to recall a word or name of a person, place, book or movie, but cannot. Often the individual will be able to retrieve some information about the word or name, such as the first letter or sound of the word or name, the number of syllables in the word or name, and sometimes words or names that sound similar to the target word or name. Brown and McNeill (1966) developed a method to elicit ToT states in the laboratory by giving participants a definition and asking them to provide the best-fitting word; this approach has been used successfully by a number of researchers to examine a variety of questions (Harley and Bown, 1998; James and Burke, 2000; Vitevitch and Sommers, 2003). ToT states have also been documented in diary studies, in which participants recorded details about each occurrence of a ToT state (Burke et al., 1991). To maintain some continuity with previous diary studies we adapted the prompts used in Burke et al. (1991; available at http://www.lcs.pomona.edu/cogaging/materials/research/TOT%20diary.pdf) for the present on-line error diary.

**FIGURE 4:**
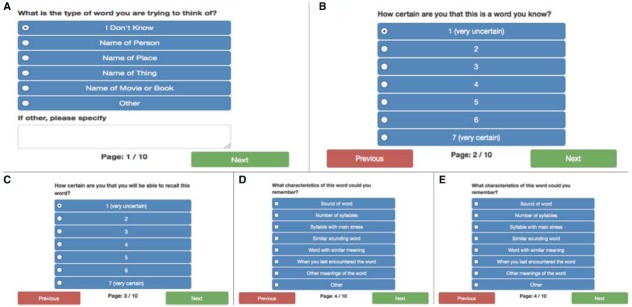
**(A)** The first screen for the ToT prompt asking for information about the type of word that cannot be recalled. **(B)** The second screen for the ToT prompt asking for information about the type of word that cannot be recalled. **(C)** The third screen for the ToT prompt asking for information about the type of word that cannot be recalled. **(D)** The fourth screen for the ToT prompt asking for information about the type of word that cannot be recalled **(E)** The fifth screen for the ToT prompt asking for information about the type of word that cannot be recalled.

The first screen for the ToT prompt asks the user to indicate which type of word cannot be recalled (Figure 4A). The second screen asks for a subjective rating on a 7-point scale “How certain are you that this is a word you know?” (Figure 4B). On the second and subsequent screens, users are free to enter as much or as little information as they recall (or wish to enter) before advancing to the next screen. The third screen asks for a subjective rating on a 7-point scale “How certain are you that you will be able to recall this word?” (Figure 4C). The fourth screen asks the user if they can recall any characteristics about the word they are trying to recall, such as the number of syllables, the first sound it starts with, etc.; multiple options can be checked here (Figure 4D). On the fifth screen the user is asked to provide any additional information they can think of about the word they are trying to recall (Figure 4E).

**FIGURE 5:**
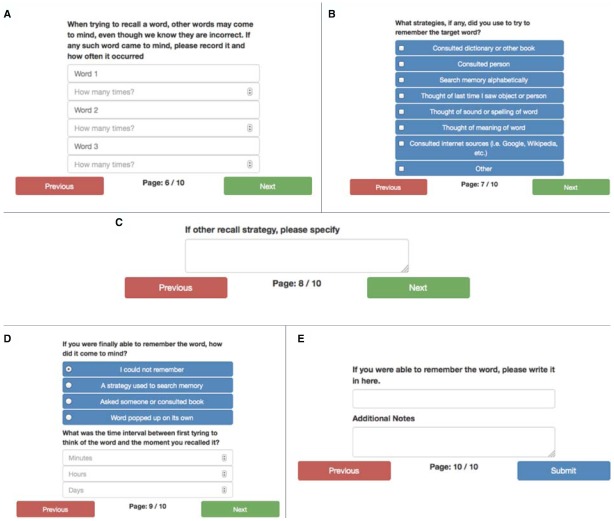
**(A)** The sixth screen for the ToT prompt asking for information about the type of word that cannot be recalled. **(B)** The seventh screen for the ToT prompt asking for information about the type of word that cannot be recalled. **(C)** The eighth screen for the ToT prompt asking for information about the type of word that cannot be recalled. **(D)** The ninth screen for the ToT prompt asking for information about the type of word that cannot be recalled. **(E)** The final screen for the ToT prompt asking for information about the type of word that cannot be recalled.

On the sixth screen (Figure 5A) the user is asked to enter any interlopers, or similar sounding words that come to mind instead of the target word. The seventh screen (Figure 5B) asks the user which strategies they may have employed to recall the name or word, such as asking a friend or consulting the Internet. Other strategies that a user might employ are asked about on the eighth page (Figure 5C). On the ninth screen, the user is asked how they were finally able to recall the work (if the ToT state was successfully resolved). They are also asked to estimate the amount of time that elapsed between the initial attempt to retrieve the word or name and the resolution of the ToT state (Figure 5D). Once the ToT has been resolved, the final screen (Figure 5E) asks the user for the word or name that produced the initial ToT state.

## Downloading the Data

All the information entered at the prompts for the on-line speech error diary (see Figures 2A and 5E) is saved in a comma-separated values (CSV) text file. This text file can be downloaded after a registered user enters his or her account name and password using the “Account Login” option. Recall that the “Quick Start” option was designed to quickly take registered users to the error prompts to facilitate the documentation of the speech error; the collected errors cannot be accessed using the “Quick Start” option.

The information provided in the data file is raw and not processed in any way. Researchers are encouraged to use available spreadsheet software, word-processing software, or custom written scripts or software to facilitate initial and subsequent processing of the raw data. We did not implement any type of search or sort functions on the website because we did not wish to impose a particular theoretical perspective on other users who may wish to analyze the data from a novel or alternative theoretical perspective. Although the raw nature of the data requires a bit of work on the part of a researcher to analyze it, the raw nature of the data ensures the longevity of the amassed data as theoretical perspectives come and go over time.

## Limitations of the SpEDi

The present on-line speech error diary is limited by the same issues, biases and concerns that limit all forms of naturalistic observation, including speech error collections and diaries (see another limitation of error collections described in Vitevitch et al., 2016). Despite these common limitations, we believe there is still much scientific and educational value to the present on-line speech error diary.

By “crowd-sourcing” the collection of various types of speech errors, we have potentially accelerated the pace of amassing a large enough number of errors to subject them to statistical analysis. The “crowd-sourcing” of error collection does, however, open the process to contributors who may lack even basic training in Linguistics and other language-related sciences. This means that subtle speech production errors, such as producing a phonetic feature not found in one’s native language, may go unnoticed or undocumented, or be documented incorrectly. Although this aspect of the on-line speech error diary may introduce or increase variability in the responses, we hope that the data processing carried out by researchers (to remove outliers, etc.) and the large number of errors amassed over time will provide the necessary statistical stability to enable researchers to observe novel and interesting patterns in the data.

Another limitation of the SpEDi is that the prompts for information are in English, which may limit the use of the error diary to English-speaking individuals. We recognize that tools like Google Translate can be used to translate the prompts on the webpages; unfortunately we cannot verify the accuracy of such translations. The use of English in the prompts is somewhat symptomatic of a larger issue in psycholinguistic research, namely, most of the research is in and about English (Vitevitch et al., 2014b). There is undoubtedly much insight into the mechanisms involved in various aspects of language processing that can be gained from studying languages other than English or by studying the use of more than one language at a time. Fortunately, the error diary allows users to enter information using any character that can be produced by a keyboard, enabling users of logographic, syllabic, and alphabetic orthographies to submit (at least certain types of) speech errors if their keyboard is set to their font of choice. This also opens up the possibility that linguistically-sophisticated users could employ characters from the International Phonetic Alphabet (IPA) to document more fine-grained errors with phonological transcription.

Finally, SpEDi only allows one to document three types of speech errors: slips of the tongue/malapropisms, slips of the ear, and ToT states. There are other types of language-related errors—such as slips of the key and slips of the finger—that language-users make, but the current form of SpEDi does not offer prompts for users to document these other types of language-related errors. There are also other types of motor or performance errors that humans make—such as reaching into a kitchen drawer to retrieve a knife, but instead one erroneously retrieves a spoon—that the current form of SpEDi does not offer prompts for users to document. Broader insight into cognition might be obtained if such motor/performance errors were considered alongside speech errors. Perhaps some of the limitations of SpEDi can be addressed by raising financial support through crowd-funding efforts to support later stages of development of SpEDi. Despite these limitations we believe the on-line speech error diary will prove to be a useful tool for language-related research and for scientific outreach efforts.

### Conflict of Interest Statement

The authors declare that the research was conducted in the absence of any commercial or financial relationships that could be construed as a potential conflict of interest.

## Footnotes

1 http://www.mpi.nl/dbmpi/cgi-bin/sedb/sperco_form4.pl
